# Histone acetylation and histone acetyltransferases show significant alterations in human abdominal aortic aneurysm

**DOI:** 10.1186/s13148-016-0169-6

**Published:** 2016-01-13

**Authors:** Yanshuo Han, Fadwa Tanios, Christian Reeps, Jian Zhang, Kristina Schwamborn, Hans-Henning Eckstein, Alma Zernecke, Jaroslav Pelisek

**Affiliations:** Department of Vascular and Endovascular Surgery, Klinikum rechts der Isar der Technische Universität München, Ismaninger Str. 22, 81675 Munich, Germany; Department of Vascular and Surgery, The First Hospital of China Medical University, Shenyang, China; Department of General Surgery, Shengjing Hospital of China Medical University, Shenyang, China; Department for Visceral, Thoracic and Vascular Surgery at the University Hospital, Technical University Dresden, Dresden, Germany; Institute of Pathology, Klinikum rechts der Isar der Technische Universität München, Munich, Germany; Institute of Experimental Biomedicine, University Hospital, University of Würzburg, Würzburg, Germany; DZHK (German Centre for Cardiovascular Research), partner site Munich Heart Alliance, Munich, Germany

**Keywords:** AAA, Epigenetics, Histone acetylation, Acetyltransferases, KAT/HAT

## Abstract

**Background:**

Epigenetic modifications may play a relevant role in the pathogenesis of human abdominal aortic aneurysm (AAA). The aim of the study was therefore to investigate histone acetylation and expression of corresponding lysine [K] histone acetyltransferases (KATs) in AAA.

**Results:**

A comparative study of AAA tissue samples (*n* = 37, open surgical intervention) and healthy aortae (*n* = 12, trauma surgery) was performed using quantitative PCR, immunohistochemistry (IHC), and Western blot. Expression of the KAT families GNAT (KAT2A, KAT2B), p300/CBP (KAT3A, KAT3B), and MYST (KAT5, KAT6A, KAT6B, KAT7, KAT8) was significantly higher in AAA than in controls (*P* ≤ 0.019). Highest expression was observed for KAT2B, KAT3A, KAT3B, and KAT6B (*P* ≤ 0.007). Expression of KAT2B significantly correlated with KAT3A, KAT3B, and KAT6B (*r* = 0.705, 0.564, and 0.528, respectively, *P* < 0.001), and KAT6B with KAT3A, KAT3B, and KAT6A (*r* = 0.407, 0.500, and 0.531, respectively, *P* < 0.05). Localization of highly expressed KAT2B, KAT3B, and KAT6B was further characterized by immunostaining. Significant correlations were observed between KAT2B with endothelial cells (ECs) (*r* = 0.486, *P* < 0.01), KAT3B with T cells and macrophages, (*r* = 0.421 and *r* = 0.351, respectively, *P* < 0.05), KAT6A with intramural ECs (*r* = 0.541, *P* < 0.001) and with a contractile phenotype of smooth muscle cells (SMCs) (*r* = 0.425, *P* < 0.01), and KAT6B with T cells (*r* = 0.553, *P* < 0.001). Furthermore, KAT2B was associated with AAA diameter (*r* = 0.382, *P* < 0.05), and KAT3B, KAT6A, and KAT6B correlated negatively with blood urea nitrogen (*r* = −0.403, −0.408, −0.478, *P* < 0.05). In addtion, acetylation of the histone substrates H3K9, H3K18 and H3K14 was increased in AAA compared to control aortae.

**Conclusions:**

Our results demonstrate that aberrant epigenetic modifications such as changes in the expression of KATs and acetylation of corresponding histones are present in AAA. These findings may provide new insight in the pathomechanism of AAA.

**Electronic supplementary material:**

The online version of this article (doi:10.1186/s13148-016-0169-6) contains supplementary material, which is available to authorized users.

## Background

In the past decades, abdominal aortic aneurysm (AAA) has been increasingly recognized as a leading cause of sudden death in men older than 65 years [1]. Despite considerable advances in surgical treatment, the only reliable diagnostic option so far is the measurement of the diameter of AAA. Exceeding 5.5 cm, patients generally undergo surgical or endovascular repair [2, 3]. Thus, a better understanding of the pathophysiologic processes leading to AAA wall destabilization until rupture remains an important issue to identify patients at increased risk.

Pathophysiological changes in gene expression of various factors within the vessel wall are the reason of many cardiovascular diseases. Among others, epigenetics have been recognized as a powerful tool to activate or silence gene transcription by changes in the chromatin structure without alterations of the DNA sequence [4]. Many factors are involved in the establishment of epigenetic traits, including DNA methylation and multitudinous modifications of histones such as methylation, acetylation, or phosphorylation [4, 5]. In particular, targeted histone alterations determine the epigenetic state of the genome. One of the most important histone modifications is attachment or removal of an acetyl group, leading either to gene activation or repression [5]. The histone acetylation process is regulated by the balanced activities of two key enzyme families of transferases, namely lysine [K] histone acetyltransferases (KATs) [6], and histone deacetylases (HDACs) [7]. The function of KATs is to add an acetyl group to the lysine residue, resulting in chromatin opening and gene activation [8]. In the context of histone acetylation, four families of KATs have been described so far (GNAT, p300/CBP, MYST, and TF-related family), comprising in total 11 acetyltransferases [9, 10].

Although the role of epigenetics as a potential mechanism to control gene activity has been proposed in cardiovascular diseases [11], few and inconsistent studies have investigated such epigenetic changes to date. For example, genomic DNA isolated from human atherosclerotic lesions was found to be hypomethylated [12]. Recently, our group demonstrated significant differences in histone and DNA methylation and the expression of corresponding methyltransferases at different stages of atherosclerosis in carotid arteries [13]. Krishna et al. have hypothesized that epigenetic mechanisms may also play a role in the pathogenesis of AAA [14]. However, epigenetics in AAA have not been addressed experimentally.

The aim of the present study was therefore to analyze the expression profiles of known KATs in AAA and healthy aortic tissue. Furthermore, we examined their main histone substrates in individual cell types within AAA. Our results provide interesting data about a possible role of specific KATs and histone acetylation in the epigenetic regulation of AAA development and progression.

## Results

### KAT mRNA expression levels and their correlations in AAA

**Fig. 1:**
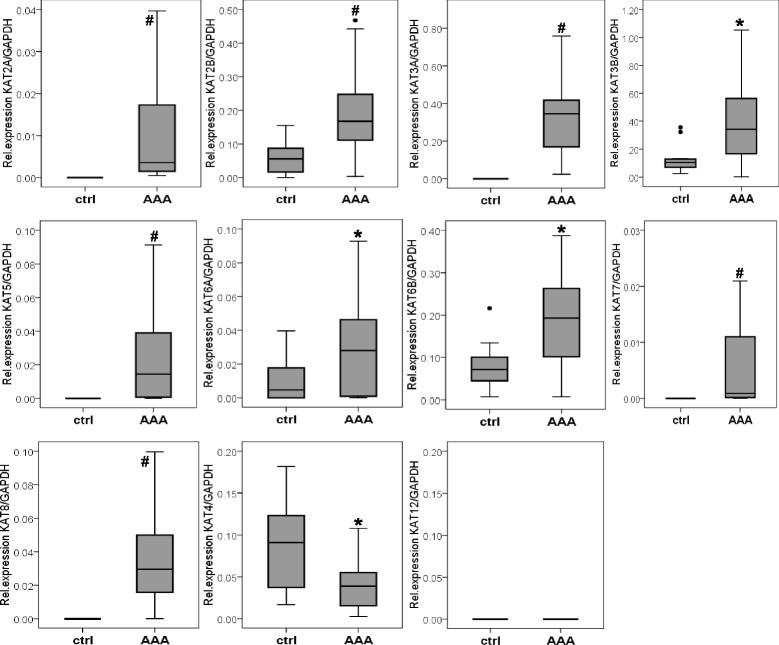
Expression analysis of lysine [K] histone acetyltransferases (KATs) in AAA and healthy aorta at mRNA level. Quantification was performed by SYBR green-based RT-PCR. Relative expression indicates expression of individual KATs related to the expression of GAPDH set as 1 (100 % expression). *AAA* specimens of abdominal aortic aneurysm (*n* = 37), *ctrl* control healthy aorta (*n* = 12). *P < 0.05, **#** *P* < 0.001

We first determined the expression of KATs in AAA at the messenger RNA (mRNA) level using quantitative real-time reverse transcriptase polymerase chain reaction (RT-PCR) and compared our results with that of control aortic tissue samples. The mRNA expression of histone acetyltransferases *KAT2A*, *KAT2B*, *KAT3A*, *KAT3B*, *KAT5*, *KAT6A*, *KAT6B*, *KAT7*, and *KAT8* belonging to the GNAT, p300/CBP, and MYST family of KATs was significantly higher in AAA than in healthy control tissue (Fig. 1). In contrast, *KAT8*, a member of the TF-related family of KATs, was detected neither in AAA nor in healthy aortic tissue. Among the above analyzed acetyltransferases, *KAT2A*, *KAT3A*, *KAT5*, *KAT7*, and *KAT8* transcripts were not detected in control aorta. In contrast, *KAT4*, belonging to the family of TF-related KATs, was significantly decreased in AAA specimens compared to controls (Fig. 1). For a better overview of the expression levels of the KATs analyzed in our study, mRNA expression levels normalized to GAPDH are depicted in Additional file 1: Table S3. The highest expression in AAA tissue was observed for *KAT2B* (2.5-fold higher in AAA compared to control aorta), *KAT3A* (not expressed in healthy aorta), *KAT3B* (3.9-fold higher in AAA), and *KAT6B* (2.8-fold higher in AAA).

KATs are frequently activated in clusters [15, 16]. We therefore in addition analyzed the inter-relationships of the expression of the individual KATs in human AAA tissue samples (Table 1). *KAT2B* correlated significantly with *KAT3A*, *KAT3B*, and *KAT6B* (*r* = 0.705, 0.564, and 0.528, *P* < 0.001 and <0.01, respectively), *KAT3A* correlated with *KAT6B* (*r* = 0.407, *P* < 0.05), *KAT3B* correlated with *KAT6B* and *KAT8* (*r* = 0.500 and 0.342, *P* < 0.01 and <0.05, respectively), *KAT5* correlated with *KAT7* and *KAT8* (*r* = 0.357 and 0.443, *P* < 0.05 and <0.01, respectively), *KAT6A* correlated with *KAT6B* (*r* = 0.532, *P* < 0.01), and *KAT4* correlated with *KAT8* (*r* = 0.648, *P* < 0.01), suggesting corporate activity especially of *KAT2B*, *KAT3A*, *KAT3B*, and *KAT6B*. Selected examples of the correlation analysis with a statistically significant outcome are depicted as dot blots in Additional file 2: Figure S1.

**Table 1:** Inter-related correlation between KATs in AAA

*r*	KAT2A	KAT2B	KAT3A	KAT3B	KAT5	KAT6A	KAT6B	KAT7	KAT8	KAT4
KAT2A	–	n.c.	n.c.	n.c.	n.c.	n.c.	n.c.	n.c.	n.c.	n.c.
KAT2B		–	0.705***	0.564**	n.c.	n.c.	0.528**	n.c.	n.c.	n.c.
KAT3A			–	n.c.	n.c.	n.c.	0.407*	n.c.	n.c.	n.c.
KAT3B				–	n.c.	n.c.	0.500**	n.c.	0.342*	n.c.
KAT5					–	n.c.	n.c.	0.357*	0.443**	n.c.
KAT6A						–	0.531**	n.c.	n.c.	n.c.
KAT6B							–	n.c.	n.c.	n.c.
KAT7								–	n.c.	n.c.
KAT8									–	0.648**
KAT4										–

Significant differences between individual KATs: *P<0.05, **P<0.01, ***P<0.001; *n.c.* no correlation

### Protein expression and cellular localization of KATs in inflammatory cells in AAA

**Fig. 2:**
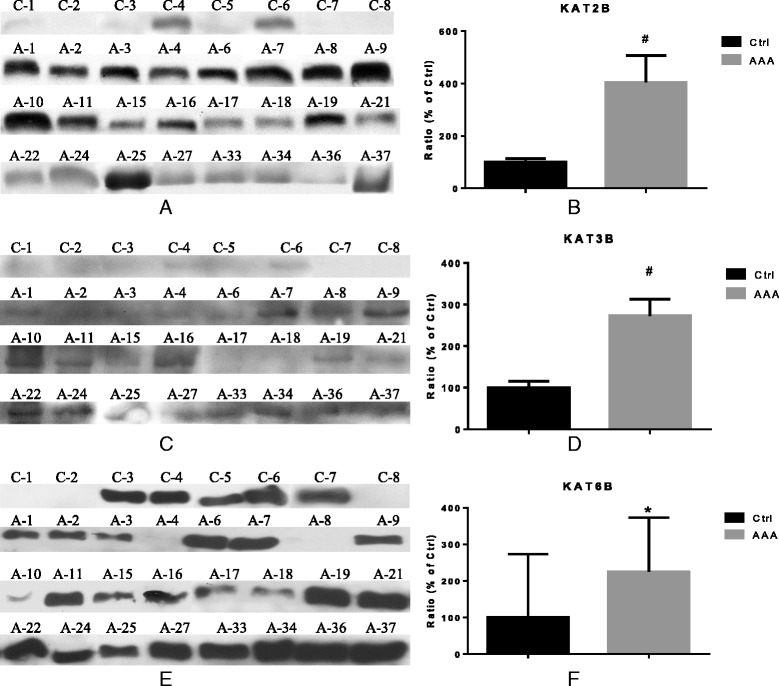
Expression analysis of KAT2B, KAT3B, and KAT6B in AAA and healthy aorta at protein level. **a**, **c**, **e** Western blot analysis. **b**, **d**, **f** Quantification of the band intensities relative to the expression of GAPDH. Ratio (% of Ctrl) indicates relative expression to Ctrl set as 100 %). *C and ctrl*, control healthy aorta (*n* = 8), *A/AAA* specimens of abdominal aortic aneurysm (*n* = 24). *P < 0.05, **#** *P* < 0.001

In order to further evaluate the protein expression of selected KATs that showed highest expression on mRNA level, Western blot analyses were performed. Corroborating our results on the mRNA level, protein expression of KAT2B, KAT3B, and KAT6B was significantly higher in AAA tissue compared to the protein expression in healthy control aortae (4.1-fold, 2.8-fold, and 2.2-fold, *P* < 0.001, <0.001, and 0.033, respectively; see Fig. 2). As we could not detect *KAT3A* mRNA expression in control tissue and no appropriate antibodies are commercially available to detect KAT3A in formalin-fixed paraffin-embedded (FFPE) tissue samples, we omitted to analyze KAT3A at the protein level.

**Fig. 3:**
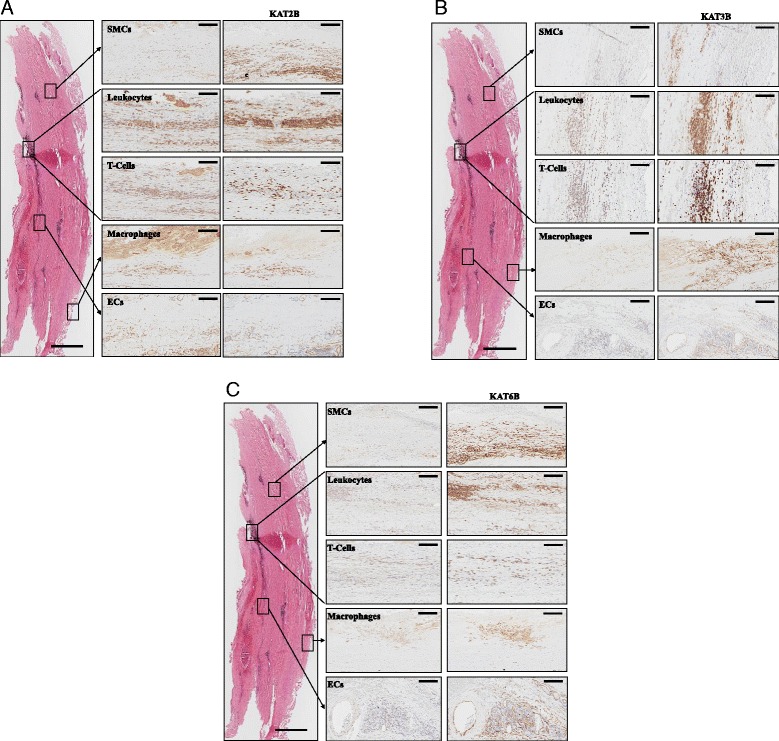
Expression analysis of KAT2B (**a**), KAT3B (**b**), and KAT6B (**c**) in AAA using IHC for cellular localization. Overview image (l*eft panel*) of the whole AAA tissue sample with areas selected for cellular localization of KAT expression (haemalum-eosin staining). The magnified images depict consecutive staining of cells as revealed by staining for indicated markers within the AAA wall and indicated KATs. *Scale bar*, overview image 1000 μm

The most common cells in AAA are smooth muscle cells (SMCs) and inflammatory cells, such as macrophages and lymphocytes [17]. We analyzed the cellular localization of KAT2B, KAT3B, and KAT6B within the AAA wall by immunohistochemistry (IHC) in consecutively stained sections. KAT2B expression was found to predominantly colocalize to CD45^+^ leukocytes, CD68^+^ macrophages, and CD3^+^ T cells (Fig. 3a). In addition, staining of intramural CD31^+/^CD34^+^ endothelial cells in neovessels was also found to colocalize with the expression of KAT2B. In contrast, an only marginal staining of KAT2B was detected in smooth muscle cells (Fig. 3a). Staining patterns of KAT3B similarly showed a strong colocalization with leucocytes and T cells but not with macrophages, medial SMCs, or neovessels (Fig. 3b). Staining for KAT6B predominantly localized to leukocytes, macrophages, and T cells, while luminal endothelial cells (ECs) and neovessels as well as SMCs did not show co-staining with this histone acetyltransferase (Fig. 3c). In contrast to the AAA tissue samples, KAT2B, KAT3B, and KAT6B could not be detected in healthy aortic tissue (data not shown). Summarizing the IHC results, our data demonstrate that KAT2B, KAT3B, and KAT6B expression is predominantly found in inflammatory cells in AAA.

### Expression of main histone substrates and their cellular source

**Fig. 4:**
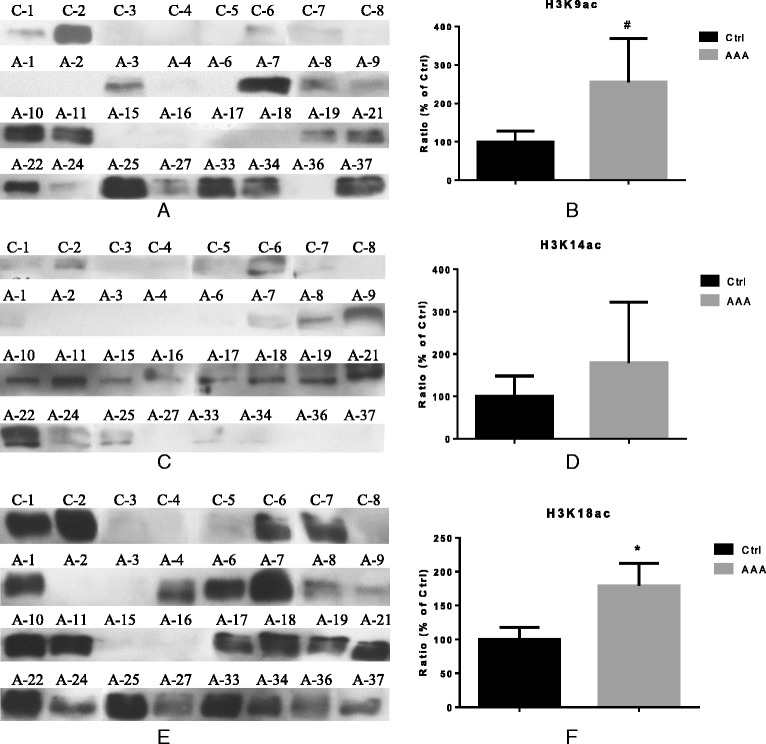
Analysis of acetylated H3K9 (H3K9ac), H3K14 (H3K14ac), and H3K18 (H3K18ac) in AAA and healthy aorta at protein level. **a**, **c**, **e** Western blot analysis. **b**, **d**, **f** Quantification of the band intensities relative to the expression of GAPDH. Ratio (% of Ctrl) indicates relative expression to Ctrl set as 100 %). *C and ctrl* control healthy aorta (*n* = 8), *A and AAA* specimens of abdominal aortic aneurysm (*n* = 24). *P < 0.05, **#** *P* < 0.001

In order to determine the acetylation of the main histone substrates of KAT2B, KAT3B, and KAT6B (Additional file 1: Table S2) [8], expression of H3K9ac, H3K14ac, and H3K18ac was determined in AAA tissue samples compared with healthy aortic tissue specimens by Western blotting. As no appropriate antibody against H3K36ac was available, analysis of this histone substrate had to be omitted. Acetylation of H3K9 (H3K9ac) and H3K18 (H3K18ac) was 2.8-fold and 1.8-fold higher in AAA than healthy aortic tissue (*P* = 0.004 and 0.019, respectively, Fig. 4a–d). Expression levels of acetylated H3K14 was 1.9-fold higher in AAA than in control aortae; however, without reaching statistical significance due to the heterogeneity of the individual values (*P* = 0.338, Fig. 4e, f).

**Fig. 5:**
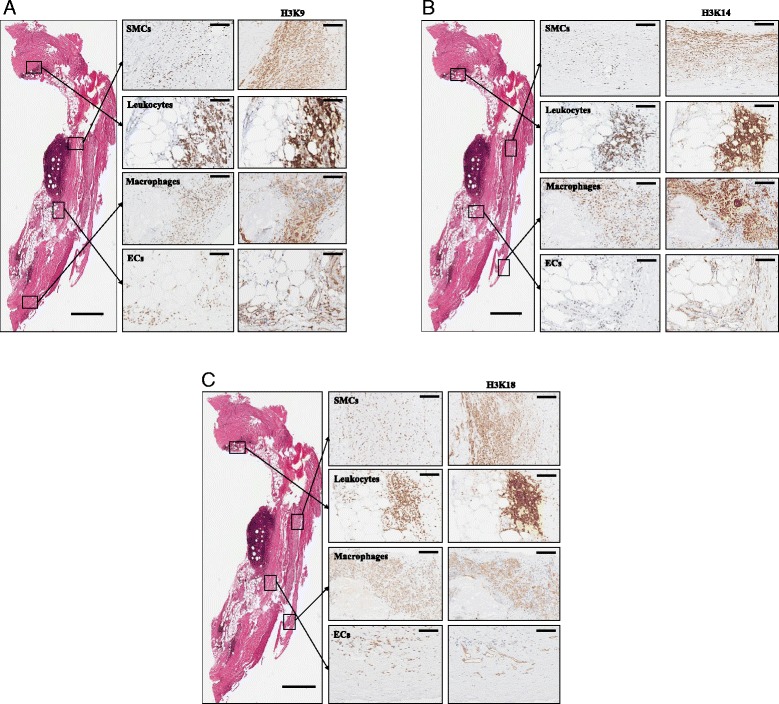
Analysis of acetylated H3K9 (**a**), H3K14 (**b**), and H3K18 (**c**) in AAA using IHC for cellular localization. Overview image (*left panel*) of the whole AAA tissue sample with areas selected for cellular localization of KAT expression (hemalum-eosin staining). The magnified images depict consecutive staining of individual cell types, as revealed by staining for indicated markers, within the AAA wall and indicated corresponding substrates. *Scale bar*, overview image 1000 μm, detailed images 100 μm

We further assessed the cellular localization of H3K9ac, H3K14ac, and H3K36ac by IHC in consecutive sections. H3K9ac staining was found to colocalize with CD45^+^ leukocytes, CD68^+^ macrophages, and CD3^+^ T cells (Fig. 5a). Furthermore, CD34^+^ and CD31^+^ neovessels were positive for acetylated H3K9 (H3K9ac). In contrast, only weak or negative staining of H3K9ac was detectable in SMCs (Fig. 5a). Similar to H3K9ac, acetylated H3K14 (H3K14ac) was mainly colocalized to CD45^+^ leukocytes, CD68^+^ macrophages, and CD34^+^/CD31^+^ neovessels. Again, staining in SMCs was very weak and not all cells were positive (Fig. 5b). Acetylation of H3K18 (H3K18ac) was most intensive in CD45^+^ leukocytes and CD68^+^ macrophages (Fig. 5c). In contrast, only some CD34^+^/CD31^+^ neovessels and SMCs were weakly positive for H3K18ac (Fig. 5c).

### Correlation analysis of KATs with cell markers

Additional experiments were performed to further confirm the localization of KATs in the individual cells within AAA observed by IHC. Because we were not able to extract individual cells from AAA tissue samples, we correlated mRNA expression of the selected KATs with markers indicative of different cell types. CD45 was selected for leukocytes, CD3 for T cells, and MSR1 for macrophages. As different phenotypes of SMCs co-exist in AAA, namely synthetic and contractile SMCs, we selected markers for both cell types, with smoothelin (SMTN) and SM myosin heavy chain (SM-MHC, MYH11) representing the contractile phenotype and SMemb/non-muscle MHCB (MYH10) and collagen I representing the synthetic phenotype [18]. Furthermore, we also analyzed the expression of vascular cell adhesion molecule (VCAM)-1, which plays an important role in the development of AAA [17]. A significant positive correlation was found for *KAT2B* with *MSR1*, indicative of macrophages, and *VCAM-1* (Table 2, Additional file 3: Figure S2A). In addition, significant correlations were observed of *KAT3B* with *CD45*, *MSR1*, and *CD3*, indicative of leukocytes, macrophages, and T cells, respectively (Table 2, Additional file 3: Figure S2B). Positive correlations were also observed for *KAT6B* with leukocyte and T cell markers (Table 2, Additional file 3: Figure S2C), further corroborating the association of KATs with inflammatory cell infiltrates. Among the expression of other KATs, *KAT3B* and *KAT5* correlated with *CD45* and *KAT6A* with *CD3*, *MYH10*, and *VCAM-1*, and a negative correlation was found for *KAT7* with *MYH11* (Table 2).

**Table 2:** Correlation between KAT expression and expression of markers of cells in AAA

*r*	CD45	CD3	MSR1	SMTN	MYH11	MYH10	Coll I	VCAM-1
KAT2A	n.c.	n.c.	n.c.	n.c.	n.c.	n.c.	–0.528***	n.c.
KAT2B	n.c.	n.c.	0.388*	n.c.	n.c.	n.c.	n.c.	0.486**
KAT3A	0.396*	n.c.	n.c.	n.c.	n.c.	n.c.	n.c.	n.c.
KAT3B	0.421*	0.361*	0.351*	n.c.	n.c.	n.c.	n.c.	n.c.
KAT5	0.378*	n.c.	n.c.	n.c.	n.c.	n.c.	n.c.	n.c.
KAT6A	n.c.	0.389*	n.c.	n.c.	n.c.	0.425**	n.c.	0.541***
KAT6B	0.609***	0.553***	n.c.	n.c.	n.c.	n.c.	n.c.	n.c.
KAT7	n.c.	n.c.	n.c.	n.c.	–0.377*	n.c.	n.c.	n.c.
KAT8	n.c.	n.c.	n.c.	n.c.	n.c.	n.c.	n.c.	n.c.
KAT4	n.c.	n.c.	n.c.	n.c.	n.c.	n.c.	n.c.	n.c.

*P<0.05, **P<0.01, ***P<0.001. *n.c.* no correlation detected

### Correlation analysis of KATs with blood parameter

We finally evaluated a possible correlation of the expression of KATs with blood parameters available for the AAA patients of our study. A summary of these correlations is provided in Additional file 1: Table S4. Interestingly, AAA diameter positively correlated with the expression of KAT2B (*r* = 0.353, *P* < 0.05). Furthermore, three different KATs, namely KAT3A, KAT6A, and KAT6B were negatively associated with the concentration of blood urea nitrogen (*r* = −0.403, −0.408, and −0.478, *P* < 0.05, *P* < 0.05, and *P* < 0.01, respectively; Additional file 4: Figure S3).

## Discussion

In the current study, we analyzed histone acetylation and the expression of corresponding histone acetyltransferases in human AAA compared to healthy aortic tissue. Our results show that members of all three families of KATs, GNAT, p300/CBP, and MYST were significantly overexpressed in the AAA wall compared to healthy aorta, particularly KAT2B, KAT3A, KAT3B, and KAT6B. These acetyltransferases were predominantly found in colocalization with macrophages and T cells, and their main histone substrates were H3K9, H3K14, and H3K18. Interestingly, some histone acetyltransferases such as KAT2B correlated also with AAA diameter and KAT3B, KAT6A, and KAT6B were associated with blood urea nitrogen. These results demonstrate for the first time that aberrant histone acetylation occurs in AAA.

Expression analyses showed that mRNA levels of members of the GNAT, CBP, and MYST family of KATs were significantly increased in the vessel wall of AAA patients compared with healthy aortae. In general, an increased expression of KATs entails an enhanced histone acetylation, and a plethora of previous studies in other tissues have provided evidence that hyperacetylated histone lysine residues are related to transcriptionally active chromatin, facilitating accessibility of the DNA template to the transcriptional machinery [19, 20]. For instance, KAT2B has been described to activate the MMP-9 promoter either independently or in a synergistic manner [21]. MMP-9 has been established as one of the key mediators of the degradation of extracellular matrix proteins in the arterial wall in AAA. Another example is KAT3A, which acetylates not only histones but also transcription factors such as p53 and thereby facilitates their binding to DNA [22]. p53 is furthermore closely associated to SMC apoptosis. Accelerated apoptosis and necrosis of SMCs, which are the main producers of extracellular matrix proteins, lead to a weakening of the aortic wall stability [23] and can entail the expression of inflammatory cytokines and proteolytic enzymes in the vessel wall, contributing to the accumulation of inflammatory cells within the AAA wall and consequently to the progression of AAA.

In contrast to KATs of the GNAT, CBP, and MYST family, the expression of members of the TF-related family was either significantly lower in AAA (KAT4) or were detected in neither AAA nor control tissue (KAT12). KAT4 is a component of the TFIID complex, which is a general transcription factor allowing RNA Pol II to bind to the promoters of protein-coding genes in living cells to initiate mRNA synthesis [24, 25]. Furthermore, the transcription factor TFIIIC has a barrier function mediated by the RNA Pol III and genome organization [26], leading to cell growth arrest and impairment of proper cellular function. The expression of KAT4 may thus be important for the maintenance of normal cellular functions in the healthy aortic tissue, which is impaired in the diseased aorta.

Interestingly, a significant inter-relationship between individual KATs in AAA determined by correlation analyses was observed in many cases, suggesting that the expression of some of these KATs is regulated in clusters. For instance, KAT6A correlated with KAT6B. This may be explained by the fact that these two KATs are highly homologous and share the same lysine substrate H3K14. In addition, KAT6A and KAT6B are promiscuous transcriptional co-activators involved in the transcriptional activation mediated by Runx1 and Runx2 that interact with these KATs [27]. Furthermore, a significant positive correlation was found between mRNA levels of KAT5 and KAT7 and of KAT5 and KAT8 in AAAs. These three KATs belong to the same family of histone acetyltransferases called MYST, show high homology, and share the same substrate histone H4 [9, 10]. However, little is known about their role in chromatin modification. The yeast NuA4 histone acetyltransferase complex, a homolog of human KAT5, is known to be involved in transcription, cell cycle control, and DNA repair [28, 29]. In this regard, the MYST family of KATs might also be involved in these processes.

A significant over-expression of KAT2B was found in the AAA wall. Recently, Bastiaansen et al. demonstrated that KAT2B acts as master switch in inflammatory processes required for effective arteriogenesis [30]. In our work, we found a significant colocalization of KAT2B with macrophages and endothelial cells of neovessels within the intima in AAA, which may suggest its association with inflammation and neovascularization in AAA development [17]. Furthermore, two important cell cycle regulators, E2F1 and p53, can interact with KAT2B [31, 32]. Transcription factor E2F1 induces S-phase-specific gene expression and is involved in promoting S-phase entry. In contrast, p53 inhibits cell cycle progression and entry in the S-phase by posttranslational protein modifications. Acetylation by KAT2B has three functional consequences on E2F1 activity: an increased DNA-binding ability and gene activation and an increase in the half-life of the protein [33]. In endothelial cells, acetylation is associated with the VEGF signaling pathway and appears to be predominantly mediated by KAT2B, and inhibition of KAT2B expression is sufficient to hinder angiogenesis [34]. These data may indicate that KAT2B can promote inflammation and neovascularization in AAA. In contrast, acetylation of the p53 region is observed after DNA damage, leading to cell cycle arrest or apoptosis [35]. So, the overexpression of KAT2B may entail growth arrest and/or apoptosis of SMCs. Interestingly, SMCs within the aortic wall were mostly negative for this acetyltransferase, suggesting protection from apoptosis and cell death in AAA.

Finally, results of our present work demonstrate that the expression of KAT2B is significantly associated with the diameter of AAA. This is an important finding, as KAT2B is a master switch in inflammation, which again is a driving force in AAA progression. In consequence, KAT2B may also be a potential biological marker of patients at increased risk of AAA rupture. Such an assertion needs to be confirmed in further studies. On the other hand, it is to mention that the expression of KAT2B negatively correlated with the amount of blood leukocytes (WBC), which was surprising, because inflammatory cells play a crucial role during development and progression of AAA [3]. However, Wilson et al. [36] demonstrated e.g., no elevation of inflammatory cells in ruptured aneurysms. The authors also suggested other mechanisms leading to the rupture of AAA. Our results showed a significant correlation between the expression of KAT2B and the macrophage marker MSR-1. These data are somewhat inconsistent and require additonal studies in the future.

Our data regarding KAT3B (p300) were contradictory. On the one hand, inflammatory cells were strongly positive for this acetyltransferase within the AAA wall, particularly in T cells. On the other hand, we found only a weak correlation of KAT3B with markers of inflammatory cells. No evidence is available of a possible role of KAT3B in cardiovascular disease. However, KAT3B and estrogen receptor (ER) function cooperate to increase the efficiency of transcription initiation [37]. Furthermore, estrogen receptor-α (ER-α) promoter methylation is increased in atherosclerotic lesions and a similar promoter methylation was found also in SMCs obtained from atheromata [38]. This may imply that KAT3B can also contribute to AAA formation.

KAT6B was identified as another highly expressed histone acetyltransferase in AAA. Here, a strong positive correlation was observed between its expression and markers of inflammatory cells, namely CD45 and CD3. KAT6B contains multiple functional domains and may be involved in both positive and negative regulation of transcription. At its C-terminus, KAT6B possesses a potent transcriptional activation domain, whereas a strong transcriptional repression domain is located at its N-terminus [39]. Consequently, KAT6B may facilitate but also inhibit processes leading to AAA formation. Thus, a potential role of KAT6B in AAA has to be further elucidated.

Interestingly, high expression of KAT2B, KAT3B, and KAT6B was found in inflammatory cells in the diseased aorta. AAA is characterized by chronic inflammation throughout the media and adventitia, which leads to the upregulation and release of multiple cytokines [40–42] and the activation of a plethora of proteolytic enzymes [2, 17], ultimately leading to a rapid expansion of AAA and rupture. In this regard, it is of great interest that several studies have already demonstrated that KAT2B and KAT3B are involved in the modulation of NF-κB activity [43] and are required to co-activate p65-dependent transcription to activate several NF-κB-regulated inflammatory genes, known to be involved in cardiovascular disease, such as eotaxin, GM-CSF (granulocyte-macrophage colony-stimulating factor), and TNFα [44]. Based on our results, inflammatory cells, particularly T-lymphocytes and macrophages, seem to be experiencing the greatest epigenetic changes in AAA. Further studies are necessary, e.g., isolating the individual cells and analyzing them separately for histone acetylation and the expression of corresponding KATs to elucidate the exact role of epigenetics in these cells relating to AAA progression and potential risk of rupture.

Some limitations of our current work should be considered. Our study comprises a relatively small sample size. Furthermore, a large variation among the values from the individual tissue specimens was observed. For this reason, we attempted to adjust our data for the total amount of cells within the AAA wall, extent of inflammation, age, diameter, hyperlipidemia, smoking, and rupture. Nevertheless, no significant correlation between the factors used for adjustment and expression of KATs was found and no improvement of our results was achieved. Furthermore, as most of our samples were formalin fixed, the cellular localization of KATs was evaluated in consecutively stained sections and indirectly by correlation analyses with specific cells markers. In addition, the analysis of epigenetic changes in inflammatory cells in AAA could not be directly compared with control healthy aortic tissue samples because these specimens do not have many inflammatory cells. Thus, the conclusion that an over-expression of KATs is found in CD45 and CD3 positive cells is based on our results in AAA, without any comparison with other inflammatory cells, e.g., from peripheral blood.

## Conclusions

Research on epigenetics is increasingly recognized to play an important role during various pathophysiological processes and diseases. Our current data provide evidence that epigenetics and chromatin modification may play an important role in AAA. As enzymatic epigenetic regulators can be altered by natural or designed compounds, their targeting may emerge as a potential novel diagnostic and therapeutic strategy in cardiovascular disease.

## Methods

### Patients and tissue collection

Samples of 37 patients (30 males, 7 females) with AAA were obtained during elective open surgical repair. All tissue samples were collected in a standardized manner from the anterior sac of the infrarenal abdominal aorta. Furthermore, all clinical data available were recorded for each patient, including age, sex, AAA diameter, hypertension, hyperlipidemia, hypercholesterolemia, chronic kidney disease, diabetes, and smoking within the preceding 6 months. Patients with Ehlers-Danlos syndrome, Marfan syndrome, and other known vascular or connective tissue disorders were excluded from the study. Aortic tissue from 12 organ donors was used as a control (7 males, 5 females), obtained from the Department of Trauma Surgery. Exclusion criteria for the control group included cancer, infection, and any other immune-related disease. Baseline characteristics of donors are summarized in Additional file 1: Table S1. The study was performed according to the Guidelines of the World Medical Association Declaration of Helsinki. The Ethics Committee of Klinikum rechts der Isar, Technische Universitaet Muenchen approved the study, and written informed consent was given by all patients.

All tissue samples were divided into two parts. The first part was fixed overnight in formalin embedded in paraffin (FFPE) and used for histological and immunohistochemical analyses or quantitative real-time reverse transcriptase-PCR (RT-PCR). The other part was immediately frozen in liquid nitrogen and used for protein extraction and quantitative Western blot analysis. The nomenclature of the investigated KATs, their alternative names, corresponding histone substrates, and proposed functions are summarized in Additional file 1: Table S2.

### RNA extraction and quantitative RT-PCR analyses (qPCR)

Total cellular RNA was isolated from FFPE sections (20 μm thickness) adjacent to the sections used for histological characterizations using the High Pure RNA Paraffin Kit according to the manufacturer’s instructions (Roche, Mannheim, Germany). The amount and the purity of RNA was determined by spectrophotometry. High-quality RNA samples used in the study had an A260/A280 ratio >1.8. For PCR analysis, RNA was reverse-transcribed into complementary DNA (cDNA) with random hexamer primers and cDNA Synthesis Kit RevertAid (Fermentas, St. Leon-Rot, Germany). Quantitative real-time RT-PCR was performed using SYBR green fluorescence dye (PeqLab, Erlangen, Germany) and StepOnePlus real-time PCR-System (Applied Biosystems/Life Technologies, Darmstadt, Germany). A modified amplification protocol was applied to eliminate bias by primer dimer using following PCR conditions: initialization step 5 min at 95 °C, denaturation 10 s at 95 °C, annealing 30 s at 60 °C, extension 10 s at 72 °C, and primer dimer elimination 15 s at 77 °C, 45 cycles.

Amplification of a housekeeping gene glyceraldehyde 3-phosphate dehydrogenase (GAPDH) was used for normalizing of the gene expression. All primers used in the study were purchased from Qiagen as designed QuantiTect Primer Assays: GAPDH (GAPDH_1), KAT2A, KA2B, KAT3B (EP300), KAT4 (TAF1), KAT5, KAT6A, KAT6B, KAT7, KAT8, KAT12; MSR-1, CD45 (PTPRC_5), CD3 (CD3D_1), SMTN, MYH10, MYH11, VCAM-1, and Collagen I (COL1A_1). The quantitative PCR analyses for all samples were independently repeated at least two times, and in the case of heterogeneous results, additional PCR was performed.

### Immunohistochemistry (IHC)

Histological and immunohistochemical analyses were performed on representative sections of aortic tissue samples (2–3 μm). Paraffin sections were routinely stained with hematoxylin-eosin (HE) and Elastica van Gieson (EvG) to assess tissue morphology, cellular composition, degree of infiltration with inflammatory cells, and the content of elastin and collagen fibers in all AAA samples. For immunohistochemistry, dewaxed and hydrated tissue sections were boiled to retrieve antigen epitopes, washed and treated with appropriate antibodies (Abs) accordingly. For analysis of cells localized within the AAA wall, smooth muscle cells were detected by primary Abs targeting smooth muscle myosin heavy chain 1 and 2 (SM-MHCII, rabbit monoclonal, dilution 1:1,000; Abcam, Cambridge, UK),^13^ endothelial cells by anti-CD31 (mouse monoclonal, dilution 1:40; Dako), and anti-CD34 (mouse monoclonal; dilution 1:400; Dako). Macrophage/monocytes were detected with anti-CD68 (mouse monoclonal, dilution 1:2000; Dako), leukocytes with anti-CD45 (mouse monoclonal, dilution 1:200; Dako), and T-lymphocytes with anti-CD3 (mouse monoclonal, dilution 1: 400; Dako). For detection of KATs, the following Abs were applied: KAT2B (rabbit polyclonal, dilution 1:200; Abcam), KAT3B (rabbit polyclonal, dilution 1:100; Abcam), and KAT6B (rabbit polyclonal, dilution 1:400; Abcam). Histone main substrates were detected with the following Abs for acetylated forms of these locations: H3K9ac (rabbit polyclonal, dilution 1:1500; Abcam), H3K14ac (rabbit monoclonal, dilution 1:1000; Abcam), and acH3K18 (rabbit polyclonal, dilution 1:1500; Abcam). All primary Abs were detected and visualized by LSAB ChemMate Detection Kit (Dako) according to the manufacturer’s instructions.

To detect the expression of KATs by immunohistochemistry and also by PCR in the specific cell types, corresponding consecutive slides were used in all cases.

### Protein extraction and Western blot analyses

Fresh frozen samples corresponding to FFPE specimen were homogenized in liquid nitrogen, suspended in lysis buffer (50 mM Tris-HCl pH 8, 150 mM NaCl, 1 % NP-40, 0.1 % sodium dodecyl sulfate, 0.5 % sodium deoxycholate, 0.02 % sodium azide), and collected by centrifugation, and the supernatants containing the cellular proteins were used for analyses. Histone extraction was carried out using EpiSeeker Histone Extraction kit (Abcam) according to the manufacturer’s instructions. Protein or histone concentrations of each specimen were determined using BCA Protein Assay Kit (Thermo Scientific, Bonn, Germany). Protein lysates and histone extracts (30 μg of each sample) were subjected to SDS-PAGE using either 7.5 or 15 % gel depending on the protein size and transferred onto polyvinylidene-difluoride (PVDF) membrane. The membranes were blocked (5 % BSA in PBS with 0.05 % Tween 20, pH 7.4) for 2 h, followed by incubation with primary antibody at a dilution of 1:500 for anti-KAT3B, 1:1000 for anti-KAT2B, anti-KAT6B, and GAPDH, overnight at 4 °C. The blots were then incubated with appropriate horseradish peroxidase conjugated secondary antibodies at a dilution of 1:1000 for 1–2 h at room temperature. Immunoreactive bands were developed using a chemiluminescence detection system (SuperSignal West Pico Chemiluminescent Substrate, Thermo Scientific, Bonn, Germany) and detected with LAS1000 (Fuji Film, Tokyo, Japan). The densitometry was performed with Image J software 1.44 (W. Rasband, Research Services Branch, NIMH, National Institutes of Health, Bethesda, MD) and normalized to the signal intensity of GAPDH for equal protein loading control of each sample in each experiment (Additional file 5: Figure S4).

### Statistical analysis

All data were analyzed using SPSS for Windows version 20.0 (SPSS Inc, Chicago, IL, USA). First, data distribution was evaluated by one-sample Kolmogorov-Smirnov test. Accordingly, continuous variables were compared by either the parametric *t* test for unpaired samples or the non-parametric Mann-Whitney U test. The data were monitored using either standard bar graphs or a box plot diagram showing median and 25th/75th percentiles. Correlations between continuous variables were quantified using Pearson’s correlation coefficient for normally distributed samples or Spearman’s rank correlation coefficient for non-parametric values. All statistical comparisons were two-sided in the sense of an exploratory data analysis using *P* < 0.05 as the level of significance.

## Availability of supporting data

The data sets supporting the results of this article are included within the article and its additional files

## Additional files

Additional file 1: Tables S1–S4.
**Table S1.** Characteristics of the study subjects. **Table S2.** Nomenclature of histone acetyltransferases used in the study. **Table S3.** Summary of expression levels of KATs analyzed in this study normalized to the expression of GAPDH. **Table S4.** Correlation between KAT expression and clinical findings of AAA patients. (DOC 84 kb) Additional file 2: Figure S1. Selected examples of scatter plot graphs from correlation analysis of inter-relationships between KAT2B and KAT3B (A), KAT2B and KAT6B (B), KAT3b and KAT6B (C) in AAA at mRNA level. Quantification was performed by SYBR green-based RT-PCR using KATs expression intensity normalized to GAPDH. AAA (*n* = 37). (PPTX 74 kb) Additional file 3: Figure S2. Selected examples of scatter plot graphs from correlation analysis between expression of KATs and specific markers of cells in AAA at mRNA level. KAT2B (A), KAT3B) (B), KAT6B (C). Quantification was performed by SYBR green-based RT-PCR. The expression of all factors was normalized to GAPDH. AAA (*n* = 37). (PPTX 152 kb) Additional file 4: Figure S3. Selected examples of scatter plot graphs from correlation analysis between expression of KATs and clinical parameters. KAT2B and AAA diameter (A), KAT3A, KAT6A, KAT6B against blood urea nitrogen (B). Quantification was performed by SYBR green-based RT-PCR. The KAT expression was normalized to GAPDH. AAA (*n* = 37). (PPTX 80 kb) Additional file 5: Figure S4. Loading controls. Expression of GAPDH at the protein level in all AAA tissue samples and all healthy aorta tissue samples (Ctrl) used in western blot analyses. (PPTX 140 kb)

## Abbreviations

AAA: aortic abdominal aneurysm
Ab: antibody
DNA: deoxyribonucleic acid
ECs: endothelial cells
EvG: Elastica van Gieson staining
FFPE: formalin-fixed paraffin-embedded
GAPDH: glyceraldehyde 3-phosphate dehydrogenase
HAT: histone acetyltransferase
HDACs: histone deacetylases
HE: hematoxylin-eosin staining
IHC: immunohistochemistry
KAT: lysine [K] histone acetyltransferase
MHC: myosin heavy chain
mRNA: messenger RNA
PVDF: polyvinylidene-difluoride
RT-PCR: real-time reverse transcriptase polymerase chain reaction
SDS-PAGE: sodium dodecyl sulfate polyacrylamide gel electrophoresis
SMCs: smooth muscle cells
