# Supplementary material for: Histone acetylation and histone acetyltransferases show significant alterations in human abdominal aortic aneurysm
Source: Clin Epigenetics. 2016 Jan 13;8:3. doi: 10.1186/s13148-016-0169-6 (PMC4711037; doi:10.1186/s13148-016-0169-6)
Supplement: Additional file 1: Tables S1–S4. — Table S1. Characteristics of the study subjects. Table S2. Nomenclature of histone acetyltransferases used in the study. Table S3. Summary of expression levels of KATs analyzed in this study normalized to the expression of GAPDH. Table S4. Correlation between KAT expression and clinical findings of AAA patients. (DOC 84 kb) [file 13148_2016_169_MOESM1_ESM.doc]

**Table S1 Characteristics of the study subjects**

|  | AAA group | Control group | P value |
| --- | --- | --- | --- |
|  | 37 | 12 |  |
|  | n (%) | n (%) |  |
| Male | 30 (81.1) | 7 (58.3) | 0.136 |
| Age (Yr) | 66.8±11.4 | 49.1±19.4 | 0.005 |
| Max AAA diameter(mm) | 62.8±16.7 | 20.2±2.9 | <0.001 |
| Hypertension | 30 (81.1) | NA | - |
| Diabetes mellitus | 1 (2.7) | NA | - |
| Hypercholesterolemia | 1 (2.7) | NA | - |
| Hyperlipidemia | 15 (40.5) | NA | - |
| Smoking | 11 (29.7) | NA | - |
| Chronic kidney disease | 1 (2.7) | NA | - |
| Rupture | 7 (18.9) | - | - |

NA = not available

**Table S2 Nomenclature of histone acetyltransferases used in the study**

| *Family* | *HAT* | *KAT** | *Histone substrate* | *Function* |
| --- | --- | --- | --- | --- |
| GNAT | Gcn5 | KAT2A | H3K9,14,18,36 | transcriptional activation,  DNA repair |
|  | PCAF | KAT2B | H3K9,14,18,36 |  |
| p300/CBP | CBP | KAT3A | H2AK5 | transcriptional activation |
|  |  |  | H2BK12,15 |  |
|  |  |  | H3K14,18 |  |
|  |  |  | H4K5,8 |  |
|  | p300 | KAT3B | H2AK5 |  |
|  |  |  | H2BK12,15 |  |
|  |  |  | H3K14,18 |  |
|  |  |  | H4K5,8 |  |
| MYST | Tip60 | KAT5 | H4K5,8,12,16 | transcriptional activation,  DNA repair,  replication |
|  | MOZ/MYST3 | KAT6A | H3K14 |  |
|  | MORF/MYST4 | KAT6B | H3K14 |  |
|  | HBO1/MYST2 | KAT7 | H4K5,8,12 |  |
|  | HMOF/MYST1 | KAT8 | H4K16 |  |
| TF-related | TAF1/TBP | KAT4 | – | transcriptional activation,  Pol III transcription |
|  | TFIIIC90 | KAT12 | H3K9,14,18 |  |

***Note**: The abbreviation used for histone acetyltransferases used in the study is KAT, derived from lysine [K] acetyltransferase.

**Table S3 Summary of expression levels of KATs analyzed in this study normalized to the expression of GAPDH**

|  | KAT2A | KAT2B | KAT3A | KAT3B | KAT5 | KAT6A | KAT6B | KAT7 | KAT8 | KAT4 | KAT12 |
| --- | --- | --- | --- | --- | --- | --- | --- | --- | --- | --- | --- |
| AAA | 0.004 | 0.167 | 0.345 | 0.343 | 0.018 | 0.028 | 0.193 | 0.002 | 0.031 | 0.039 | undet |
| Ctrl | undet | 0.066 | undet | 0.089 | undet | 0.018 | 0.069 | undet | undet | 0.068 | undet |
| **P* | <0.001 | <0.001 | <0.001 | 0.007 | <0.001 | 0.019 | 0.003 | <0.001 | <0.001 | 0.002 | 1.000 |

**Note:** **P*-values show statistical differences in expression of KATs between AAA tissue samples and controls using non-parametric Man-Withey-U-test. undet – expression was not detected.

**Table S4 Correlation between KAT expression and clinical findings of AAA patients**

| *r* | hsCRP | Urea | Cr. | CK | WBC | RBC | PLT | Diameter |
| --- | --- | --- | --- | --- | --- | --- | --- | --- |
| KAT2A | n.c. | n.c. | n.c. | n.c. | n.c. | n.c. | n.c. | n.c. |
| KAT2B | n.c. | n.c. | n.c. | n.c. | -0.359* | n.c. | n.c. | +0.353* |
| KAT3A | n.c. | -0.403* | n.c. | n.c. | n.c. | n.c. | n.c. | n.c. |
| KAT3B | n.c. | n.c. | n.c. | n.c. | n.c. | n.c. | n.c. | n.c. |
| KAT5 | n.c. | n.c. | n.c. | n.c. | n.c. | n.c. | n.c. | n.c. |
| KAT6A | n.c. | -0.408* | n.c. | n.c. | n.c. | n.c. | -0.401* | n.c. |
| KAT6B | n.c. | -0.478** | n.c. | n.c. | n.c. | n.c. | n.c. | n.c. |
| KAT7 | n.c. | n.c. | n.c. | n.c. | +0.339* | -0.362* | n.c. | n.c. |
| KAT8 | n.c. | n.c. | n.c. | n.c. | n.c. | n.c. | n.c. | n.c. |
| KAT4 | n.c. | n.c. | n.c. | n.c. | n.c. | n.c. | n.c. | n.c. |

**Note:** hsCRP = high sensitive C-reactive protein, Cr. = Creatinine, CK = Creatine kinase,

WBC = white blood cell count, RBC = red blood cell count, PLT = platelets
