# Supplementary figures and images for: Histone acetylation and histone acetyltransferases show significant alterations in human abdominal aortic aneurysm
Source: Clin Epigenetics. 2016 Jan 13;8:3. doi: 10.1186/s13148-016-0169-6 (PMC4711037; doi:10.1186/s13148-016-0169-6)

## Slide 1
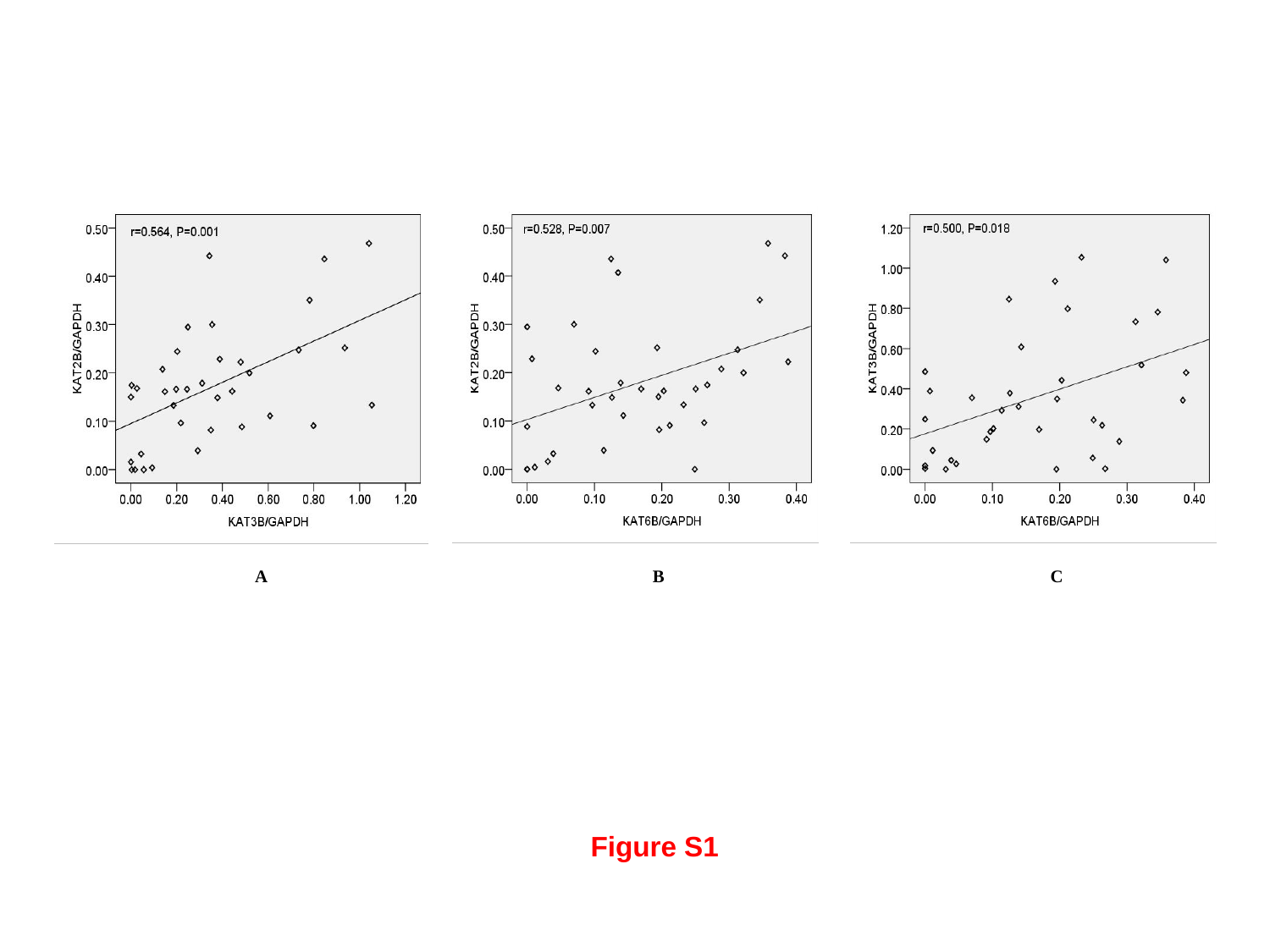

Supplement: Additional file 2: Figure S1. — Selected examples of scatter plot graphs from correlation analysis of inter-relationships between KAT2B and KAT3B (A), KAT2B and KAT6B (B), KAT3b and KAT6B (C) in AAA at mRNA level. Quantification was performed by SYBR green-based RT-PCR using KATs expression intensity normalized to GAPDH. AAA (n = 37). (PPTX 74 kb) [file 13148_2016_169_MOESM2_ESM.pptx]

## Slide 1
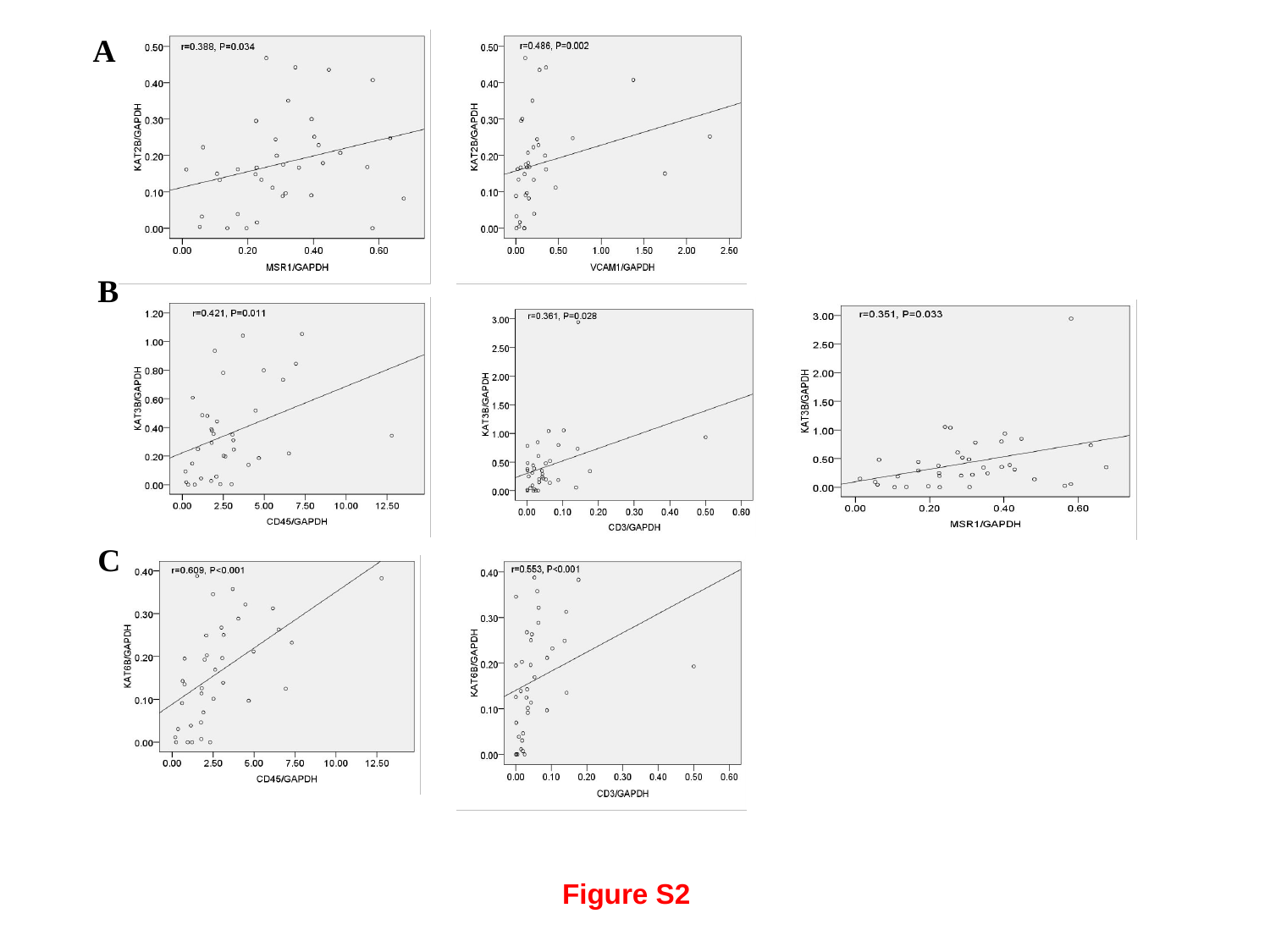

Supplement: Additional file 3: Figure S2. — Selected examples of scatter plot graphs from correlation analysis between expression of KATs and specific markers of cells in AAA at mRNA level. KAT2B (A), KAT3B) (B), KAT6B (C). Quantification was performed by SYBR green-based RT-PCR. The expression of all factors was normalized to GAPDH. AAA (n = 37). (PPTX 152 kb) [file 13148_2016_169_MOESM3_ESM.pptx]

## Slide 1
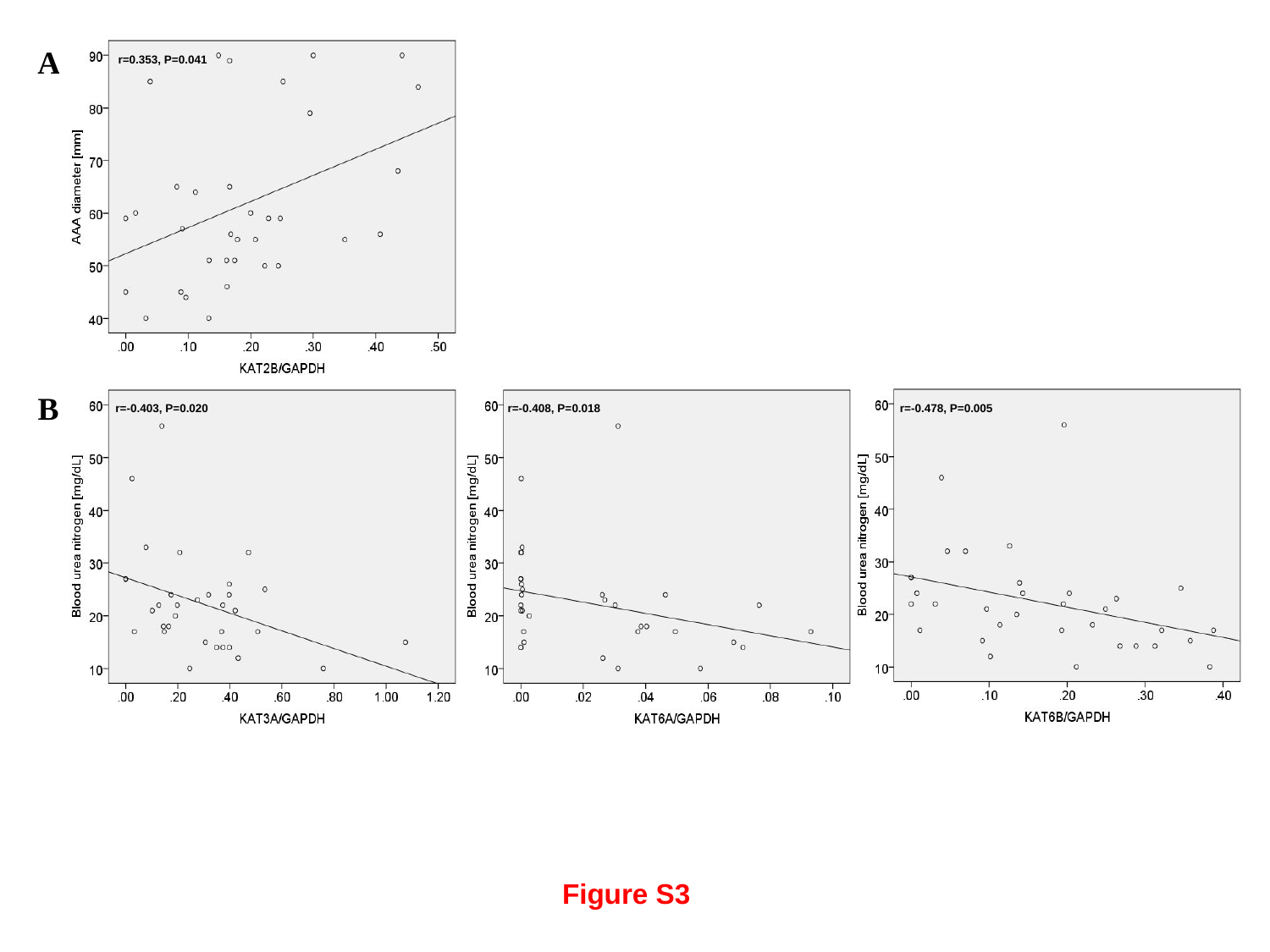

Supplement: Additional file 4: Figure S3. — Selected examples of scatter plot graphs from correlation analysis between expression of KATs and clinical parameters. KAT2B and AAA diameter (A), KAT3A, KAT6A, KAT6B against blood urea nitrogen (B). Quantification was performed by SYBR green-based RT-PCR. The KAT expression was normalized to GAPDH. AAA (n = 37). (PPTX 80 kb) [file 13148_2016_169_MOESM4_ESM.pptx]

## Slide 1
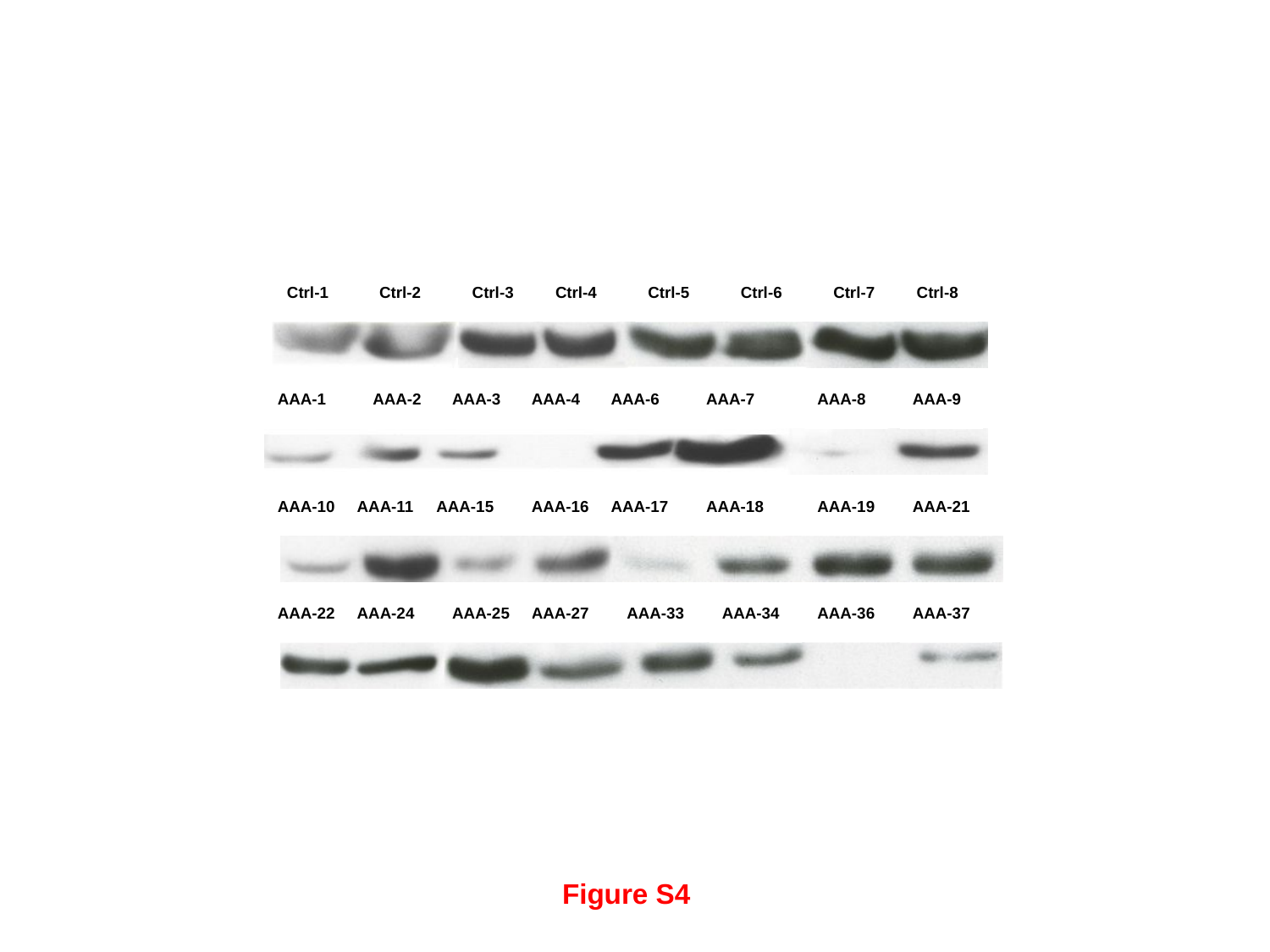

Supplement: Additional file 5: Figure S4. — Loading controls. Expression of GAPDH at the protein level in all AAA tissue samples and all healthy aorta tissue samples (Ctrl) used in western blot analyses. (PPTX 140 kb) [file 13148_2016_169_MOESM5_ESM.pptx]
